# The Impact of Rhodiola Rosea Extract on Strength Performance in Alternative Bench-Press and Bench-Pull Exercises Under Resting and Mental Fatigue Conditions: A Randomized, Triple-Blinded, Placebo-Controlled, Crossover Trial

**DOI:** 10.3390/nu17060940

**Published:** 2025-03-07

**Authors:** Daniel Marcos-Frutos, Žiga Leban, Zhaoqian Li, Xing Zhang, Paula M. Lara, Carlos Alix-Fages, Pablo Jiménez-Martínez, Nadia Zebboudji, Annabelle Caillet, Beatriz Redondo, Jesús Vera, Danica Janicijevic, Amador García-Ramos

**Affiliations:** 1 Department of Physical Education and Sport, Faculty of Sport Sciences, University of Granada, 18071 Granada, Spain; 97220417@student.upr.si (Ž.L.); lzqf3ng@gmail.com (Z.L.); starz@correo.ugr.es (X.Z.); c.alix@icen.es (C.A.-F.); p.jimenez@icen.es (P.J.-M.); n.zebboudji@pharmanager-ingredients.com (N.Z.); a.caillet@pharmanager-ingredients.com (A.C.); amagr@ugr.es (A.G.-R.); 2 Faculty of Health Sciences, University of Primorska, 6310 Izola, Slovenia; 3 CLARO (Clinical and Laboratory Applications of Research in Optometry) Laboratory, Department of Optics, Faculty of Sciences, University of Granada, Campus de La Fuentenueva 2, 18001 Granada, Spain; paulamlv.pl@gmail.com (P.M.L.); beatrizrc@ugr.es (B.R.); veraj@ugr.es (J.V.); 4 Department of Health Research, ICEN Canary University, 38002 Santa Cruz, Spain; 5 R&D Department Pharmanager Ingredients, 49100 Angers, France; 6 Faculty of Sports Science, Ningbo University, Ningbo 315211, China; jan.danica@gmail.com; 7 Department of Radiology, Ningbo No. 2 Hospital, Ningbo 315010, China; 8 Department of Sports Sciences and Physical Conditioning, Faculty of Education, Universidad Católica de la Santísima Concepción, Concepción 4070129, Chile

**Keywords:** adaptogens, exhaustion, ergogenic aids, resistance training, muscle endurance

## Abstract

**Objectives**: This study aimed to explore the effects of four days of Rhodiola Rosea (RR) supplementation on bench-press and bench-pull exercises under resting or mental fatigue conditions in young healthy individuals. **Methods**: Eighteen participants (seven women) visited the laboratory on five occasions separated by 7 days—one preliminary session and four experimental sessions. In the preliminary session, participants were familiarised with the Stroop and Multiple Object Tracking tests, after which their one-repetition maximum loads for bench presses and bench pulls were determined. The four experimental sessions had the same protocol, differing only in the supplement (RR or placebo) and mental task conditions (Stroop test or control video). Participants were assigned randomly and counterbalanced to each experimental condition: (I) RR and Stroop test, (II) RR and control video, (III) placebo and Stroop test, and (IV) placebo and control video. **Results**: The main findings indicate that RR supplementation has trivial-to-small effects in terms of mental fatigue, visuo-cognitive processing, or perceived exertion. However, RR was significantly superior to placebo on strength performance in the control video condition during some sets, as it increased the number of repetitions performed in the bench press and the fastest velocity in the bench pull. Out of 52 comparisons, 17 small effect sizes were observed, with 14 favouring RR and 3 favouring placebo, with the remaining differences being trivial. **Conclusions**: These results suggest that short-term RR supplementation is safe and provides its main ergogenic effects on physical performance rather than in visuo-cognitive or mental outcomes.

## 1. Introduction

Mental fatigue is commonly defined as a gradual, cumulative psychobiological state caused by demanding cognitive tasks, characterised by physiological, behavioural, and subjective symptoms such as a lack of energy and tiredness [1,2,3]. Mental fatigue can impair cognitive performance during working activities [4], driving [5], or studying [6]. The negative effects of mental fatigue extend to physical tasks, particularly during endurance activities, with reported decrements in time to exhaustion during both cycling [7] and submaximal isometric contractions [1,8]. Therefore, it is not surprising that researchers are continuously looking for strategies to counter mental fatigue, either to prevent its onset (e.g., caffeine ingestion) or to aid recovery after it has occurred (e.g., listening to music) [9]. One of the most practical and accessible options for mitigating the occurrence of mental fatigue is the strategic use of nutritional supplements [9,10]. Several nutritional supplements have been shown to reduce behavioural or subjective markers of mental fatigue, including caffeine, creatine, panax ginseng, and various vitamins or minerals [9,10]. Among these, Rhodiola Rosea (RR), a supplement with purported adaptogenic and ergogenic properties, has gained attention [11].

Rhodiola Rosea is hypothesised to enhance mental fatigue and physical performance by potentially upregulating acutely catecholamine levels (i.e., norepinephrine and epinephrine), which are key neurotransmitters and hormones in the stress response [12]. Catecholamines, released via the activation of the sympathoadrenal system, increase alertness and cognitive processing speed by enhancing cerebral blood flow, neuronal excitability, and glucose availability to neurons, thereby alleviating mental fatigue and improving tasks like the Stroop test [13,14]. Simultaneously, an increase in catecholamines, such as epinephrine and norepinephrine, enhances physical performance by activating the sympathoadrenal system, which stimulates glycogenolysis and lipolysis in skeletal muscle [15]. This may rapidly increase ATP and glucose availability, providing energy for more forceful and sustained muscle contractions [16]. Additionally, catecholamines heighten calcium sensitivity in muscle fibres and boost neuromuscular transmission, improving motor unit recruitment and contractile efficiency [17,18]. These effects may counteract the detrimental effects of mental fatigue on physical performance [1,14,19]. Theoretically, these effects are underpinned by Rhodiola Rosea’s adaptogenic modulation of the hypothalamic–pituitary–adrenal axis and monoamine pathways [20].

In humans, several randomised controlled trials have reported reductions in mental fatigue after RR supplementation without adverse effects [21,22,23]. For instance, Spasov et al. [21] found that 20 days of 100 mg of RR improved subjective markers of mental fatigue and general well-being compared to placebo. Darbinyan et al. [22] demonstrated that 170 mg daily for 14 days reduced mental fatigue during stressful night shifts. Similarly, Shevtsov et al. [23] showed that both 370 mg and 555 mg doses could reduce mental fatigue after a single intake, with no dose-dependent differences. While two of these studies also reported improvements in cognitive performance on several tests compared to placebo [22,23], Spasov et al. [21] did not observe such improvements.

In addition to mental fatigue, RR has been linked to improved endurance performance [24,25]. Noreen et al. [25] reported a shorter 6-mile cycling time trial after a single dose of 3 mg·kg^−1^ of RR without changes in ratings of perceived exertion (RPEs). De Bock et al. [24] found that 200 mg of RR taken one hour before exercise improved time to exhaustion during cycling, although no ergogenic effects were observed after 14 days of supplementation. However, other studies have failed to observe improvements in time to exhaustion during endurance tasks [26,27,28]. Williams et al. [12] conducted the only study that examined the impact of RR on resistance training performance. They supplemented 1500 mg of RR daily for three days and reported improvements in the fastest set velocity during the bench press exercise performed at 75% of one repetition maximum (1RM), but paradoxically the total number of repetitions completed during the three subsequent sets to failure was compromised. Therefore, there is a need for more rigorously designed trials to explore the efficacy of RR [29], especially during resistance training.

To the best of our knowledge, this is the first triple-blinded, placebo-controlled, crossover study with the aim of exploring the effects of four days of Rhodiola Rosea Extract (RR) supplementation on strength exercise and visuo-cognitive performance in young healthy individuals. We hypothesised that RR supplementation would reduce mental fatigue, leading to improved performance on the Stroop test, and enhanced visuo-cognitive processing, as measured by the Multiple Object Tracking (MOT) test before and after strength training. Additionally, the anticipated reduction in mental fatigue with RR supplementation was expected to increase strength training work capacity and lower RPEs.

## 2. Materials and Methods

### 2.1. Experimental Protocol

**Figure 1:**
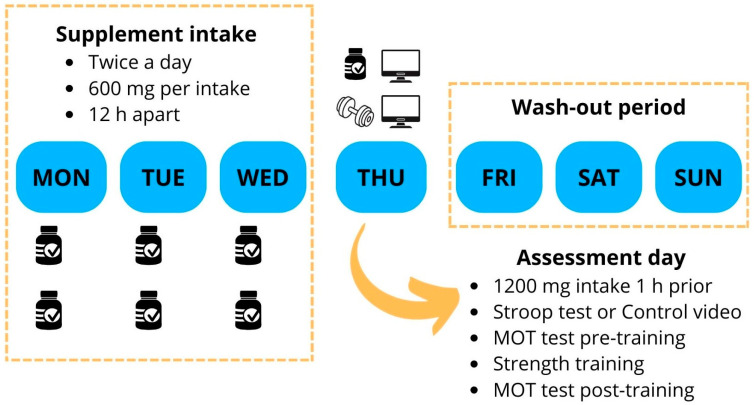
Descriptive illustration of weekly experimental procedures. MOT: Multiple Object Tracking.

The current study was a randomised, placebo-controlled, triple-blinded (participants, researchers, and data analysts), and crossover experimental trial. Participants visited the laboratory on five occasions separated by seven days: one preliminary testing session and four experimental sessions. In the preliminary testing session, participants were first introduced to the Stroop and MOT tests, after which their 1RM loads for the bench-press and bench-pull exercises were determined. The four experimental sessions had the same protocol, differing only in the supplement (RR or placebo) and mental task conditions (Stroop test or control video). Participants were counterbalanced and assigned to the following experimental session conditions using the Research Randomizer online software (www.randomizer.org) (accessed on 21 March 2024): (I) RR and Stroop test, (II) RR and control video, (III) placebo and Stroop test, and (IV) placebo and control video. The assessment order in each experimental session was as follows: (I) mental task condition (Stroop test or control video), (II) MOT pre-training, (III) strength training, and (IV) MOT post-training. All sessions were conducted on the same day of the week and at consistent times (±1 h) for individual participants (Figure 1). Participants were instructed to avoid stimulants on the testing day and refrain from resistance exercise 24 h before the visit. The CONSORT checklist is provided in Supplementary File S1.

### 2.2. Participants

Eighteen resistance-trained volunteers participated in this study (Table 1). This sample size was justified through a priori power analysis using G*Power (V.3.1.9.7, Franz Faul, Universität Kiel, Kiel, Germany). We used a target effect size (ES) of f = 1.07 from a previous study [12], with an alpha of 0.05 and power of 0.95. The analysis indicated that 14 participants were needed. The inclusion criteria required participants to be 18–40 years old, have had at least one year of resistance training experience, and be able to execute the bench-press and bench-pull exercises at maximal intentional velocity with proper technique. Data from two participants were excluded from the Stroop test analyses due to technological issues. This study adhered to the Declaration of Helsinki, it was approved by the Research Ethics Committee (SICEIA) of the Andalusian Regional Government (SICEIA-2024-000283), and it was registered on ClinicalTrials.gov (NCT06853600). All participants provided written informed consent after being briefed on the study’s objectives and experimental procedures.

### 2.3. Procedures

#### 2.3.1. Supplementation

The supplement condition involved either RR or a placebo, both administered in pill form. The pills were contained in numbered packages (1 and 2) to ensure blinding. Each RR pill contained 300 mg of RR (RhodioZen, Pharmanager Ingredients, Angers, France) standardised at 3% of rosavines and 1% of salidroses according to the manufacturer analyses, while each placebo pill contained 300 mg of maltodextrin with excipients (Life Pro Nutrition, Madrid, Spain). Participants took two pills twice daily, 12 h apart, for three days before each experimental session. On the assessment day, they consumed four additional pills one hour before arriving at the laboratory. Therefore, the daily dose was 1200 mg for four consecutive days. A 3-day washout period followed each assessment before supplement intake resumed. The supplements were prepared and packaged by a researcher not involved in the study at a separate facility (Life Pro Nutrition Industries, Madrid, Spain). The company that prepared the packages ensured the content of the products with an external certification of analysis. The contents of each package were unknown until all statistical analyses were completed. All supplements were identical in appearance, taste, and colour, and the participants were not able to distinguish between them.

#### 2.3.2. RM Load Assessment

The session began with a standardised warm-up, encompassing 5 min of light-intensity activities (jogging, cycling, or rowing) and 10 min of upper-body dynamic mobility. Bench-press and bench-pull 1RM loads were determined using free weights. A linear position transducer was used to measure the mean velocity (MV) of the barbell and to assist in load increment decisions (GymAware RS; Kinetic Performance Technologies, Canberra, Australia). Participants were encouraged to perform all repetitions at maximal intentional velocity. For both exercises, the incremental test began with 20 kg, and the load was progressively increased until failure, similar to previous studies [30,31]. For light loads (MV > 0.80 m·s^−1^ for bench press and 1.00 m·s^−1^ for bench pull), 3 repetitions were performed with 15 kg load increments. For moderate loads (MV = 0.80–0.60 m·s^−1^ for bench press and 1.00–0.80 m·s^−1^ for bench pull), 2 repetitions were performed with 10 kg load increments. For heavy loads (MV < 0.60 m·s^−1^ for bench press and < 0.80 m·s^−1^ for bench pull), 1 repetition was performed at load increments of 5 kg or less until the participant failed to lift the next load, with 1RM defined as the highest successfully lifted load. A rest period of 3 min was established between different loads for both exercises.

#### 2.3.3. Cognitively Demanding and Control Tasks

A 30 min incongruent Stroop task was used to induce mental fatigue, which requires response inhibition and sustained attention [32] and has been demonstrated to be a valid intervention for inducing mental fatigue and impairing physical performance [1,33]. In the incongruent Stroop colour word task, participants were shown lists of colour words (e.g., “blue”, “red”, “yellow”, or “green”) on the screen, where the ink colour of the letters did not match the word itself (for example, the word “blue” might be displayed in green ink). For each word, participants were instructed to select the ink colour rather than reading the word. After each response, a new word was presented. The control condition consisted of watching an emotionally neutral documentary during 30 min in the same screen (LS27C330GAUXEN, 27”, Samsung, Seoul, Republic of Korea) used for the Stroop task. Participants remained seated in the same room and in the same position for both interventions (50 cm from the screen).

#### 2.3.4. MOT Test

In line with the method used by Vera et al. [34] for the MOT test, eight identical black balls (each with a diameter of 1.8 cm) were displayed on a white square background (32.5 cm, luminance of 107 cd/m^2^) subtending a visual angle of 36°. The stimuli were presented on a 27-inch television monitor (LS27C330GAUXEN) positioned 50 cm away from the participant. At the beginning of each trial, three of the balls were randomly illuminated in green for 2 s, after which they reverted to their original black colour. Participants were instructed to track the selected balls for a duration of 10 s. No specific strategy was suggested for completing the task, and participants were allowed to use natural eye movements, mirroring real-world scenarios [35,36]. None of the participants had prior experience with the MOT task. The balls moved along linear paths at a constant speed, changing direction only when colliding with each other or the walls.

**Figure 2:**
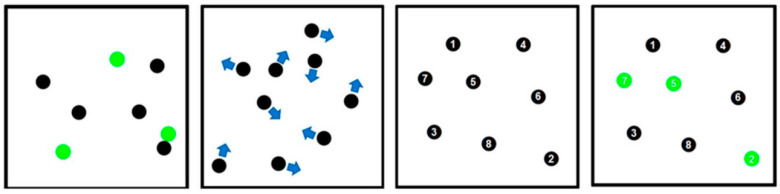
The four stages of the Multiple Object Tracking task (from left to right): the presentation stage, where three out of the eight balls were briefly highlighted in green for 2 s; the movement stage, during which all the balls returned to black and moved for 10 s, colliding and bouncing off each other; the identification stage, where the balls were frozen in place and numbered, and participants were asked to select the three balls that had originally been highlighted; and finally, the feedback stage, where participants were informed of the correct target balls.

After the 10 s tracking period, all the balls stopped, and each was labelled with a number from 1 to 8. The participant then identified the final positions of the three originally highlighted balls by selecting the corresponding numbers displayed (Figure 2). In this study, ball speed was adjusted using a 1-up, 1-down staircase method [37], where the speed increased if all three balls were correctly identified and decreased if any ball was incorrectly identified. The initial speed was set at 13.5 cm·s^−1^, and for each correct or incorrect response, the speed was increased or decreased by 1.3 cm·s^−1^, respectively. The staircase procedure ended after six reversals, and the mean speed of the last four reversals was used as the final MOT performance measure.

#### 2.3.5. Strength Training

The general warm-up mirrored that of the 1RM assessment. Thereafter, the specific warm-up included 2 sets of 3 repetitions at 30% and 50% of 1RM and a final set of 2 repetitions at 70% of 1RM for each exercise. The training regimen consisted of 8 alternating sets (4 sets each) of bench presses and bench pulls, with a 2 min rest period between sets, performed at 70% of the 1RM load established during the preliminary testing session. Sets were terminated when 2 consecutive repetitions were performed at an MV below 0.35 m·s^−1^ for the bench press and 0.60 m·s^−1^ for the bench pull. Participants were encouraged to perform all repetitions at maximal intent, and they received real-time MV feedback (GymAware RS; Kinetic Performance Technologies, Canberra, Australia). Strength performance was assessed by recording the number of repetitions completed (N_rep_) and the velocity of the fastest repetition (MV_fastest_) in each set.

The OMNI-Resistance Exercise Scale (OMNI-RES) of perceived exertion was used as a measure of perceptual fatigue [38]. Participants reported their general (G-RPE) and local RPE (L-RPE) related to the whole body and the exercising muscles, respectively. Both were reported 2 min after finishing the last training set using the OMNI-RES scale (0–10), where 0 is extremely easy, and 10 is extremely hard. An image of the OMNI-RES scale was shown to the participants.

### 2.4. Statistical Analysis

Data normality and randomness were assessed using the Shapiro–Wilk’s test and Wald–Wolfowitz runs test, respectively. To facilitate Stroop test data analysis, the 30 min of evaluation were trimmed in intervals of 5 min. A two-factor repeated-measures analysis of variance (ANOVA) test was used to examine the effects of “supplement condition” (RR vs. placebo) and “time” (0–5 vs. 5–10 vs. 10–15 vs. 15–20 vs. 20–25 vs. 25–30 min) on the percentage of correct answers and reaction time during the Stroop test. A three-factor repeated-measures ANOVA test was used to examine the effects of “supplement condition” (RR vs. placebo), “mental task condition” (Stroop test vs. control video) and “time” (pre- vs. post-training) on visuo-cognitive processing. A three-factor repeated-measures ANOVA test was used to explore the effects of “supplement condition”, “mental task condition” and “set number” (1 vs. 2 vs. 3 vs. 4) on N_rep_ and MV_fastest_. The Wilcoxon test was used to compare G-RPE and L-RPE between the “supplement condition” separately for each “mental task condition”. The factor sex was not considered in the ANOVAs because it failed to reveal any significant interaction effect for any dependent variable. Post hoc analyses with Bonferroni adjustments and Hedges’ g effect size (ES) with 95% confidence intervals were used to explore the differences between the supplement conditions. ES magnitudes were classified as follows: trivial (<0.20), small (0.20–0.59), moderate (0.60–1.19), large (1.20–2.00), and extremely large (>2.00) [39]. All analyses were conducted using SPSS software version 23.0 (SPSS Inc., Chicago, IL, USA). Statistical significance was accepted at an alpha level of 0.05.

## 3. Results

**Figure 3:**
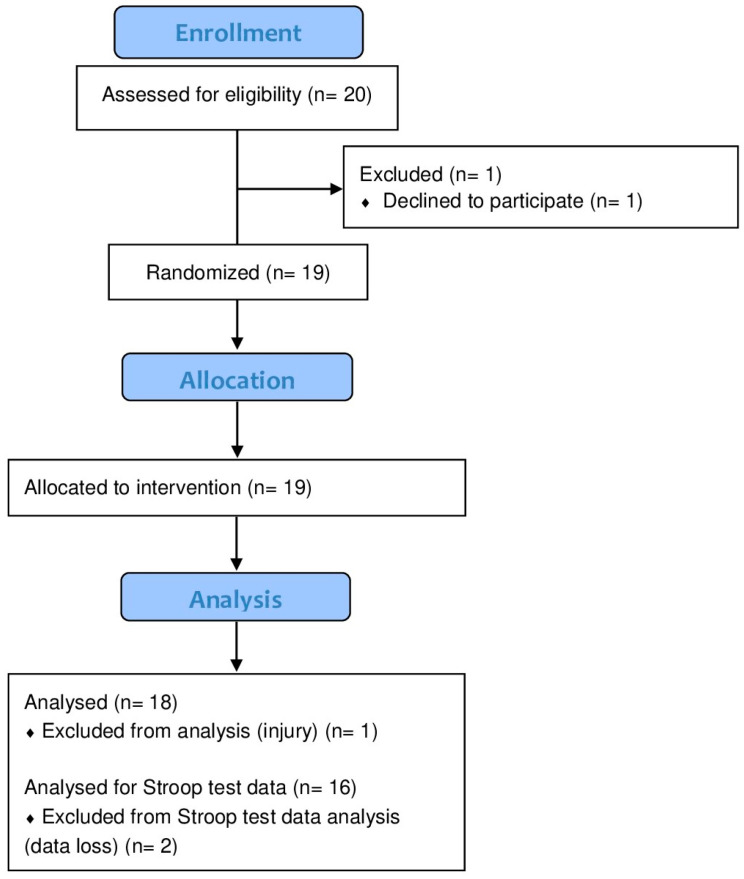
CONSORT flow diagram showing participant flow through the study.

Participant flow through this study is presented in Figure 3. Both supplements (RR and placebo) were well tolerated, and no side effects were reported with the ingestion of any of the two conditions. Due to data loss in the Stroop test dataset (n = 16), a post hoc power analysis was conducted using G*Power. Based on our results, we used an ES of *f* = 0.18. The analysis revealed a statistical power of 0.81.

### 3.1. Mental Fatigue

The main effect of “time” was significant for the percentage of correct answers, since it tended to be lower at the end of the task compared to the beginning (Table 2). However, no significant main effect of “supplement condition” or the “supplement condition” × “time” interaction was observed. Post hoc analyses further revealed no significant differences between the RR and placebo conditions throughout the 30 min Stroop test for any dependent variable. The analysis of the magnitude of the differences between the supplement conditions revealed trivial differences for 7 out of 12 comparisons (ES < 0.20), while for 5 out of 12 comparisons small differences were noted in favour of the RR condition (ES = 0.28–0.42).

### 3.2. Visuo-Cognitive Processing

No significant main effects, interactions, or post hoc differences between the supplement conditions were found for visuo-cognitive processing in the MOT test (Table 3). The analysis of the magnitude of the differences between the supplement conditions revealed trivial differences for two out of four comparisons (ES < 0.20), while for the remaining two comparisons small differences were noted in favour of the RR condition (ES = 0.37–0.38).

### 3.3. Strength Performance

The main effect of the “set number” was significant for all strength variables and exercises, indicating increased fatigue (i.e., reduction in N_rep_ and MV_fastest_) along the training session (Table 4). The main effect of the “mental task condition” was significant only for N_rep_ in the bench press exercise, as participants performed more repetitions after watching the control video compared to after completing the Stroop test. Concerning the main effect of the supplement itself, statistically significant differences were not reached for any variable or exercise. Nevertheless, a significant triple interaction (*p* = 0.007) was observed for N_rep_ in the bench press, indicating that participants completed more repetitions using RR than placebo in the first two sets of the control video condition. None of the remaining interactions including the “supplement condition” reached statistical significance. Additionally, post hoc analyses revealed significantly greater strength performance for RR compared to placebo in 4 out of 32 comparisons, while no significant differences between RR and placebo were observed for the remaining comparisons. The analysis of the magnitude of the differences between the supplement conditions revealed trivial differences for 22 out of 32 comparisons (ES < 0.20), small differences in favour of RR for 7 comparisons (ES = 0.22–0.51), and small differences in favour of placebo for 3 comparisons (ES = 0.27–0.41).

### 3.4. Perceived Exertion

Neither G-RPE nor L-RPE showed significant differences between the RR and placebo conditions regardless of the mental task condition (Table 5).

## 4. Discussion

To the best of our knowledge, this is the first triple-blinded, placebo-controlled, crossover study examining RR’s effects on strength performance under mental fatigue. The main findings indicate that RR supplementation did not differ from placebo in terms of mental fatigue, visuo-cognitive processing, or RPE. However, RR improved performance in the initial sets after watching a control video, specifically for N_rep_ in the bench-press and MV_fastest_ in the bench-pull exercises.

### 4.1. Mental Fatigue

The execution of the Stroop test for 30 min has been shown to effectively induce mental fatigue, as evidenced by subjective questionnaires [40,41]. This is in line with the progressive decline in the percentage of correct answers and the increased reaction time reported in our study, suggesting that mental fatigue led to a deterioration in cognitive performance. However, the Stroop task did not compromise subsequent visuo-cognitive performance compared to watching an emotionally neutral documentary. Similarly, no significant differences were found between the RR and placebo conditions in any Stroop test variable. These findings align with those of Spasov et al. [21] who also observed no improvement in cognitive performance after RR supplementation during mental fatigue. In contrast, other studies have reported enhanced cognitive performance with RR compared to placebo under similar conditions [22,23]. Notably, these studies assessed mental fatigue using both subjective questionnaires and reductions in cognitive performance, although they employed different cognitive tests to induce mental fatigue, and the decline in performance during the test was used to verify its effectiveness, which differs from the methods used in prior research. To better understand these conflicting results, future studies should aim to replicate the same cognitive tests across studies to enable clearer comparisons of RR’s effects on mental fatigue and cognitive performance.

### 4.2. Visuo-Cognitive Processing

This is the first study to examine the effects of RR on performance during the MOT test. Our results showed no differences between RR and placebo conditions either before or after training in the MOT test. Similarly, two other studies found no significant differences between RR and placebo groups in various cognitive processing tests [21,42]. However, two other studies reported improved cognitive performance for the RR group [22,23]. Our study utilised a daily dose of 1200 mg, which is higher than those generally used in previous studies, although the intervention duration (four days) was shorter. For instance, Spasov et al. [21] used 100 mg for 20 days, and Cropley et al. [42] administered 400 mg for 14 days. While these results suggest that longer intervention periods could be needed to induce ergogenic effects on cognitive processing, Shevtsov et al. [23] reported positive effects after a single intake of 370 mg and 555 mg doses. These conflicting results suggest that further research is needed to clarify the impact of RR on cognitive performance, especially considering that we conducted the first crossover study design.

### 4.3. Strength Performance

The only significant positive effects for RR were noted in the initial sets after watching a control video, specifically for N_rep_ in the bench presses and MV_fastest_ in the bench pulls. This suggests that RR may be more effective under basal conditions, in a non-stressed state, and could be classified as an adaptogen rather than a traditional nootropic. The reason underlying these effects could be related to the lack of ability of RR to counteract the mental fatigue induced by the Stroop test. The magnitude of the differences ranged from trivial (22 out of 32 comparisons) to small. From the 10 comparisons showing small differences (ES > 0.20), 7 favoured the RR condition, and 3 favoured the placebo condition. The comparable strength performance during strength training aligns with findings from other chronic supplementation studies [24,26,27,28].

It is worth noting that the performance benefits reported in other studies were observed in participants who were recreationally active and who were tested after taking a single dose of RR supplementation [24,25]. For instance, improvements in endurance performance were found by De Bock et al. [24] (0.4 min in a cycle ergometer test) and Noreen et al. [25] (0.4 min in a 6-mile time trial). In contrast, studies involving chronic supplementation, including our own, have consistently failed to show significant performance improvements. This suggests that any ergogenic effects of RR on physical performance may be more pronounced in acute use.

### 4.4. Perceived Exertion

No differences were observed between RR and placebo in RPE after eight alternating sets of bench-press and bench-pull exercises. Although the exercise modality differs, these results align with those from Walker et al. [28] and Noreen et al. [25] after cycling time-to-exhaustion tests and 6-mile time trials, respectively. This suggests that RR has no significant effect on perceived exertion across various forms of endurance and resistance training tasks.

### 4.5. Limitations and Directions for Future Research

While this triple-blinded, placebo-controlled, crossover study fills an important gap in understanding the effects of RR supplementation on mental fatigue, visuo-cognitive processing, strength performance, and perceived exertion, it is not without limitations. Firstly, the small sample size (18 participants, with 16 for Stroop test variables due to data loss) may have limited the statistical power to detect subtle effects. While 17 comparisons showed small effect sizes (14 favouring RR and 3 favouring placebo), only 4 of these reached statistical significance, suggesting that some observed trends may not have been strong enough to reach significance given the sample size. Secondly, the Stroop test is a widely accepted tool for inducing and measuring mental fatigue [40,41]. However, studies investigating the effects of RR supplementation have used different cognitive tasks to induce mental fatigue, such as night duties or cognitive tests [21,22,23], and have used different cognitive tests to measure this mental fatigue. These differences in mental fatigue induction and measurement complicates direct comparisons between studies. Future research should aim to recruit larger sample sizes, standardise the cognitive tests used to measure mental fatigue, and assess the concurrent use of RR supplementation and strength training on neuromuscular adaptations. Furthermore, due to the previous health research and the current ergogenic impact on physical performance, future research should address the effects of RR on vitality and physical well-being in larger and more diverse populations.

## 5. Conclusions

The present study aimed to investigate the effects of RR supplementation on strength exercise and visuo-cognitive performance. The results of this study indicate that RR supplementation has trivial-to-small effects compared to placebo in terms of mental fatigue, visuo-cognitive processing, or perceived exertion. However, RR was significantly superior to placebo on strength performance in the control video condition during some sets, as it increased the number of repetitions performed in the bench press and the fastest velocity in the bench pull, although the effects of RR were small. Overall, the findings of this study suggest that, under specific protocols, RR supplementation could provide a safe and small-to-trivial acute ergogenic effect mainly on physical rather than cognitive domains.

## Figures and Tables

**Table 1:** Descriptive data of participants.

Variables	Male (n = 11)	Female (n = 7)
Age (years)	22.8 ± 2.1	21.9 ± 4.1
Body mass (kg)	76.9 ± 4.5	62.1 ± 16.6
Height (cm)	176.8 ± 6.8	163.1 ± 9.7
Bench press 1RM/Body mass	1.14 ± 0.17	0.72 ± 0.16
Bench pull 1RM/Body mass	1.13 ± 0.19	0.85 ± 0.16

Data are reported as means and standard deviations. 1RM: 1-repetition maximum.

**Table 2:** Two-way repeated-measures ANOVA comparing Stroop test outcomes (n = 16).

Variable	Time Interval	Supplement Condition		ES
		RR	Placebo	
Correct Answers (%)	0–5 min	97.0 ± 2.2	96.8 ± 4.4	0.07 (−0.47, 0.62)
	5–10 min	96.4 ± 2.1	96.3 ± 3.3	0.00 (−0.41, 0.42)
	10–15 min	96.7 ± 1.8	95.7 ± 4.5	**0.29 (−0.25, 0.85)**
	15–20 min	95.8 ± 3.1	95.6 ± 3.6	0.06 (−0.16, 0.29)
	20–25 min	96.5 ± 2.1	96.0 ± 3.0	0.17 (−0.22, 0.58)
	25–30 min	95.6 ± 2.9	95.7 ± 2.9	−0.04 (−0.35, 0.27)
ANOVA				
Suppl: F = 0.5; *p* = 0.508		**Time:** **F = 3.8; *p* = 0.004**		Suppl × Time: F = 0.6; *p* = 0.736
Reaction Time (s)	0–5 min	0.86 ± 0.15	0.93 ± 0.25	**−0.33 (−0.86, 0.17)**
	5–10 min	0.84 ± 0.14	0.92 ± 0.22	**−0.42 (−0.96, 0.08)**
	10–15 min	0.90 ± 0.30	0.93 ± 0.23	−0.11 (−0.63, 0.40)
	15–20 min	0.87 ± 0.22	0.96 ± 0.26	**−0.34 (−0.77, 0.06)**
	20–25 min	0.85 ± 0.17	0.91 ± 0.24	**−0.28 (−0.73, 0.16)**
	25–30 min	0.88 ± 0.20	0.88 ± 0.21	−0.02 (−0.34, 0.30)
ANOVA				
Suppl: F = 2.3; *p* = 0.146		Time: F = 0.6; *p* = 0.533		Suppl × Time: F = 0.7; *p* = 0.305

Data are reported as means and standard deviations. Effect sizes are depicted with 95% confidence intervals. RR: Rhodiola Rosea; ES: effect size; Suppl: supplement condition. Bold values indicate *p* < 0.05 for ANOVA main effects and interactions, *t*-test *p*-value < 0.05, or |ES| ≥ 0.20.

**Table 3:** Three-way repeated-measures ANOVA comparing mean speed (cm·s^−1^) during visuo-cognitive processing based on the Multiple Object Tracking (MOT) test (n = 18).

Mental Task Condition	Time	Supplement Condition		ES
		RR	Placebo	
Control video	Pre	16.2 ± 3.2	16.1 ± 3.2	0.01 (−0.62, 0.63)
	Post	17.2 ± 3.6	16.1 ± 2.0	**0.38 (−0.21, 1.00)**
Stroop	Pre	16.7 ± 3.0	15.6 ± 2.6	**0.37 (−0.13, 0.90)**
	Post	16.8 ± 3.2	16.3 ± 2.6	0.16 (−0.34, 0.67)
ANOVA				
Main effect		Interaction		
Suppl: F = 1.7; *p* = 0.212 Task: F < 0.1; *p* = 0.892 Time: F = 2.6; *p* = 0.124		Suppl × Task: F < 0.1; *p* = 0.794 Suppl × Time: F = 0.2; *p* = 0.651 Task × Time: F < 0.1; *p* = 0.766 Suppl × Task × Time: F = 0.9; *p* = 0.346		

Data are reported as means and standard deviations. Effect sizes are depicted with 95% confidence intervals. RR: Rhodiola Rosea; ES: effect size; Suppl: supplement condition; Task: mental task condition. Bold values indicate *p* < 0.05 for ANOVA main effects and interactions, *t*-test *p*-value < 0.05, or |ES| ≥ 0.20.

**Table 4:** Three-way repeated-measures ANOVA comparing mechanical performance (n = 18).

Exercise	Variable	Mental Task	Set	Supplement Condition		ES
				RR	Placebo	
Bench press	N_rep_	Control video	1	**15.5 ± 3.3**	14.3 ± 2.5 *	**0.39 (0.03, 0.78)**
			2	**12.2 ± 2.5**	10.9 ± 2.3 *	**0.50 (0.12, 0.91)**
			3	9.5 ± 2.4	9.3 ± 1.3	0.08 (−0.42, 0.59)
			4	8.4 ± 2.7	8.4 ± 2.0	0.00 (−0.27, 0.27)
		Stroop	1	13.3 ± 2.9	13.6 ± 2.7	−0.10 (−0.48, 0.28)
			2	11.3 ± 2.3	11.6 ± 2.1	−0.12 (−0.50, 0.26)
			3	9.7 ± 2.3	9.2 ± 2.2	0.19 (−0.26, 0.65)
			4	8.5 ± 2.3	8.1 ± 2.5	0.16 (−0.17, 0.49)
ANOVA						
Main effect			Interaction			
Suppl: F = 2.0; *p* = 0.171 **Task: F = 4.7; *p* = 0.044** **Set: F = 91.0; *p* < 0.001**			Suppl × Task: F = 2.4; *p* = 0.142 Suppl × Set: F = 0.2; *p* = 0.863 **Task × Set: F = 7.6; *p* = 0.027** **Suppl × Task × Set: F = 4.5; *p* = 0.007**			
Bench press	MV_fastest_ (m·s^−1^)	Control video	1	0.65 ± 0.08	0.63 ± 0.07	**0.28 (−0.06, 0.63)**
			2	0.59 ± 0.05	0.60 ± 0.06	−0.08 (−0.47, 0.31)
			3	0.57 ± 0.06	0.56 ± 0.07	0.17 (−0.19, 0.53)
			4	0.54 ± 0.04	0.54 ± 0.05	−0.14 (−0.42, 0.13)
		Stroop	1	0.62 ± 0.06	0.63 ± 0.06	−0.10 (−0.58, 0.37)
			2	0.59 ± 0.06	0.58 ± 0.04	0.15 (−0.43, 0.74)
			3	0.54 ± 0.05	0.56 ± 0.05	**−0.28 (−0.74, 0.15)**
			4	0.53 ± 0.06	0.52 ± 0.05	0.17 (−0.20, 0.55)
ANOVA						
Main effect			Interaction			
Suppl: F = 0.1; *p* = 0.734 Task: F = 3.1; *p* = 0.094 **Set: F = 86.2; *p* < 0.001**			Suppl × Task: F = 0.3; *p* = 0.616 Suppl × Set: F = 0.4; *p* = 0.723 Task × Set: F = 0.1; *p* = 0.930 Suppl × Task × Set: F = 2.8; *p* = 0.091			
Bench pull	N_rep_	Control video	1	13.1 ± 4.5	12.6 ± 4.5	0.11 (−0.16, 0.38)
			2	10.6 ± 3.9	10.6 ± 3.4	−0.01 (−0.24, 0.21)
			3	10.0 ± 2.5	9.5 ± 3.4	0.16 (−0.21, 0.54)
			4	9.1 ± 2.6	9.3 ± 3.8	−0.08 (−0.57, 0.40)
		Stroop	1	12.9 ± 2.7	13.0 ± 5.1	−0.03 (−0.52, 0.47)
			2	11.7 ± 3.7	11.4 ± 5.1	0.07 (−0.31, 0.45)
			3	10.6 ± 2.4	10.4 ± 3.8	0.07 (−0.42, 0.56)
			4	9.1 ± 2.3	9.0 ± 3.4	0.04 (−0.40, 0.47)
ANOVA						
Main effect			Interaction			
Suppl: F < 0.1; *p* = 0.763 Task: F = 1.2; *p* = 0.293 **Set: F = 42.2; *p* < 0.001**			Suppl × Task: F < 0.1; *p* = 0.965 Suppl × Set: F = 0.2; *p* = 0.869 Task × Set: F = 2.1; *p* = 0.116 Suppl × Task × Set: F = 0.5; *p* = 0.694			
Bench pull	MV_fastest_ (m·s^−1^)	Control video	1	**0.78 ± 0.04**	0.76 ± 0.05 *	**0.51 (0.00, 1.05)**
			2	0.77 ± 0.06	0.75 ± 0.05	**0.37 (−0.04, 0.81)**
			3	**0.76 ± 0.05**	0.73 ± 0.04 *	**0.61 (0.07, 1.19)**
			4	0.74 ± 0.05	0.73 ± 0.06	0.17 (−0.16, 0.53)
		Stroop	1	0.80 ± 0.07	0.79 ± 0.07	0.05 (−0.52, 0.61)
			2	0.77 ± 0.06	0.76 ± 0.06	**0.22 (−0.46, 0.91)**
			3	0.74 ± 0.04	0.76 ± 0.05	**−0.41 (−1.08, 0.24)**
			4	0.72 ± 0.05	0.73 ± 0.04	**−0.27 (−0.97, 0.42)**
ANOVA						
Main effect			Interaction			
Suppl: F = 1.2; *p* = 0.281 Task: F = 1.0; *p* = 0.326 **Set: F = 31.7; *p* < 0.001**			Suppl × Task: F = 2.1; *p* = 0.167 Suppl × Set: F = 1.1; *p* = 0.349 **Task × Set: F = 4.0; *p* = 0.013** Suppl × Task × Set: F = 1.4; *p* = 0.262			

Data are reported as means and standard deviations. Effect sizes are depicted with 95% confidence intervals. N_rep_: Number of repetitions completed; MV_fastest_: Mean velocity of the fastest repetition; RR: Rhodiola Rosea; ES: effect size; Suppl: supplement condition, Task: mental task condition; * significantly different compared to RR. Bold values indicate *p* < 0.05 for ANOVA main effects and interactions, *t*-test *p*-value < 0.05, or |ES| ≥ 0.20.

**Table 5:** Wilcoxon test results comparing G-RPE and L-RPE between the supplement conditions (n = 18).

Variable	Mental Task Condition	Supplement Condition		*p*
		RR	Placebo	
G-RPE	Control video	6.8 ± 1.0	6.9 ± 1.5	0.630
	Stroop	6.7 ± 1.6	6.6 ± 1.6	0.935
L-RPE	Control video	7.4 ± 1.0	7.3 ± 1.5	0.755
	Stroop	7.1 ± 1.6	6.8 ± 1.2	0.598

Data are reported as means and standard deviations. G-RPE: general rate of perceived exertion; L-RPE: local rate of perceived exertion; RR: Rhodiola Rosea.

## Data Availability

The data and materials used to support the findings of the study are available via the following link: https://osf.io/unrfw/?view_only=3d8a2717eb704b5c983156dbb6ce07db, accessed on 23 December 2024.

## Supplementary Materials

The following supporting information can be downloaded at https://www.mdpi.com/article/10.3390/nu17060940/s1, File S1: CONSORT 2010 checklist of information to include when reporting a randomised trial.

## Abbreviations

The following abbreviations are used in this manuscript:

1RM	1-repetition maximum.
ANOVA	Analysis of variance.
G-RPE	General ratings of perceived exertion.
L-RPE	Local ratings of perceived exertion.
MOT	Multiple Object Tracking.
MV	Mean velocity.
MVfastest	Mean velocity of the fastest repetition.
Nrep	Number of repetitions completed.
OMNI-RES	OMNI-Resistance Exercise Scale.
RPEs	Ratings of perceived exertion.
RR	Rhodiola Rosea.
